# Unraveling the Multifaceted Role of TRIM21 in Virus-Triggered Innate Immunity and Diseases

**DOI:** 10.3390/biom16091237

**Published:** 2026-08-26

**Authors:** Shijin Lan, Ying Wang, Yutong Fu, Shixing Yang, Quan Shen, Xiaochun Wang, Wen Zhang, Deqiang Wang, Likai Ji

**Affiliations:** 1 Cancer Center, Affiliated Hospital of Jiangsu University, Zhenjiang 212013, China; 2212413102@stmail.ujs.edu.cn; 2 School of Clinical Medicine and Medical Laboratory Science, Jiangsu University, Zhenjiang 212013, China; 3 School of Basic Medicine and Public Health, Jiangsu University, Zhenjiang 212013, China

**Keywords:** TRIM21, innate immunity, ubiquitination, selective autophagy, viruses

## Abstract

Tripartite motif-containing protein 21 (TRIM21/Ro52) is a pivotal E3 ubiquitin ligase and cytoplasmic fragment crystallizable receptor (FcR). It plays a crucial role in viral infections, autoimmune disorders, and cancers by regulating multiple cell signaling axes, including the NF-κB, RIG-I-like receptor (RLR), cGAS-STING, and Toll-like receptor (TLR) pathways. Type I interferon (IFN-I), a pleiotropic cytokine, is produced via these immune signaling pathways, which are often triggered by viral components. TRIM21 both activates IFN-I signaling and mediates its negative feedback through post-translational modification of key immune signaling proteins, thereby maintaining immune homeostasis. In recent years, TRIM21 has been found to dually regulate autophagy and IFN-I in the context of virus–host interplay. Herein, we systematically summarize the functional roles of TRIM21 in virus-triggered intracellular immunity, aiming to provide insights for researchers and inspire further investigation in this area.

## 1. Introduction

Several classes of pattern recognition receptors (PRRs), including retinoic acid-inducible gene I (RIG-I)-like receptors (RLRs), Toll-like receptors (TLRs), and DNA sensors, have been identified as key sensors of DNA or RNA viruses, initiating innate antiviral immune responses. In RNA virus-infected cells, RIG-I and MDA5 recognize viral RNA and signal through MAVS to activate TBK1/IKKε and IRF3, thereby inducing production of type I interferon (IFN-I). This RLR signaling cascade is tightly controlled by post-translational modifications, particularly ubiquitination [1,2]. Additionally, endosome-localized TLR3, TLR7, and TLR8 sense viral single-stranded RNA (ssRNA) and trigger TRIF- or MyD88-dependent pathways, respectively, leading to IRF3 activation and subsequent IFN-I production. TLRs have emerged as not only key initiators of innate antiviral immunity but also as promising therapeutic targets, underscoring the importance of molecules that fine-tune TLR-mediated signaling [3]. For DNA viruses, the cyclic GMP-AMP synthase cGAS and TLR9 serve as major cytosolic sensors for double-stranded DNA (dsDNA) and unmethylated CpG DNA, respectively. Upon activation, cGAS produces 2′3′-cGAMP, which in turn activates stimulator of interferon genes (STING) to orchestrate innate immune responses through the cGAS-STING-TBK1/IKKε-IRF3 axis [4].

**Figure 1:**
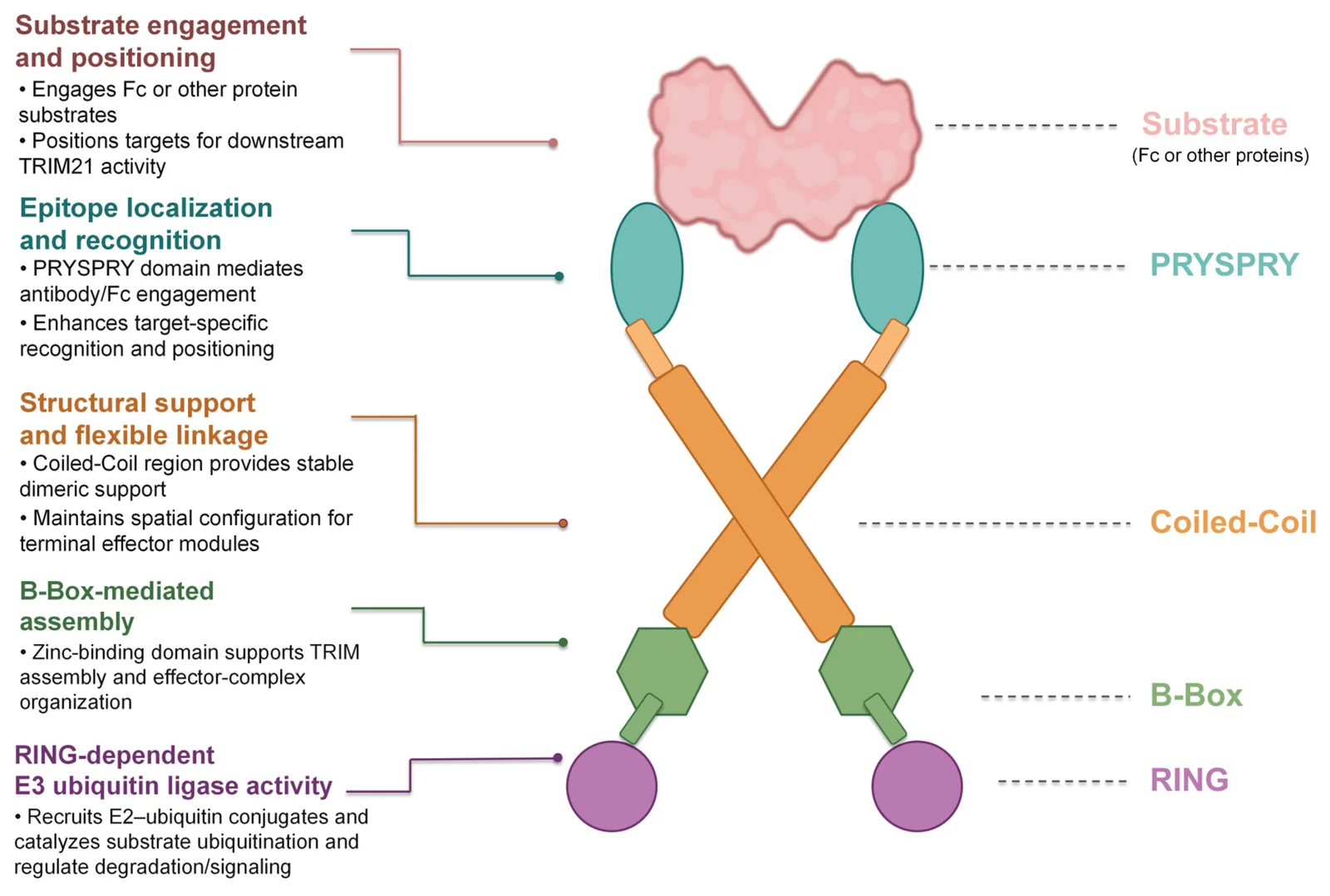
Schematic representation of TRIM21 domain architecture and functional organization. The figure was created using Microsoft PowerPoint (Microsoft Office 365).

TRIM21 (also known as Ro52) is a cytosolic member of the TRIM family and an Fc receptor (FcR), playing a crucial role in pathogen-associated diseases. As a member of the tripartite motif (TRIM) protein family, which comprises approximately 77 members in humans [5], TRIM21 shares the characteristic structural organization of TRIM proteins (Figure 1). Most TRIM proteins contain a conserved N-terminal RBCC motif consisting of a RING finger domain, one or two B-box domains, and a coiled-coil region [6]. The RING domain confers E3 ubiquitin ligase activity, whereas the B-box and coiled-coil regions contribute to protein–protein interactions, dimerization, and higher-order oligomerization [7]. Through this conserved structure–function framework, TRIM proteins, including TRIM21, participate in diverse biological processes such as immune regulation, signal transduction, cellular differentiation, DNA damage responses, and tumorigenesis [5]. In antiviral immunity, these properties enable TRIM21 to regulate the stability, activity, and subcellular fate of key host and viral proteins through ubiquitination-dependent and -independent mechanisms, thereby influencing virus sensing, signal amplification, and pathogen clearance [8,9]. In addition, TRIM21 possesses the distinctive ability to recognize antibody-bound intracellular pathogens through its Fc-binding activity, further linking humoral recognition to intracellular immune defense. Recent studies have uncovered multiple regulatory mechanisms by which TRIM21 interacts with diverse proteins to modulate their activity in virus-infected cells. In this review, we systematically summarize the functional roles of TRIM21 in virus-induced intracellular immunity.

## 2. TRIM21 Regulates NF-κB Complex Activity

**Figure 2:**
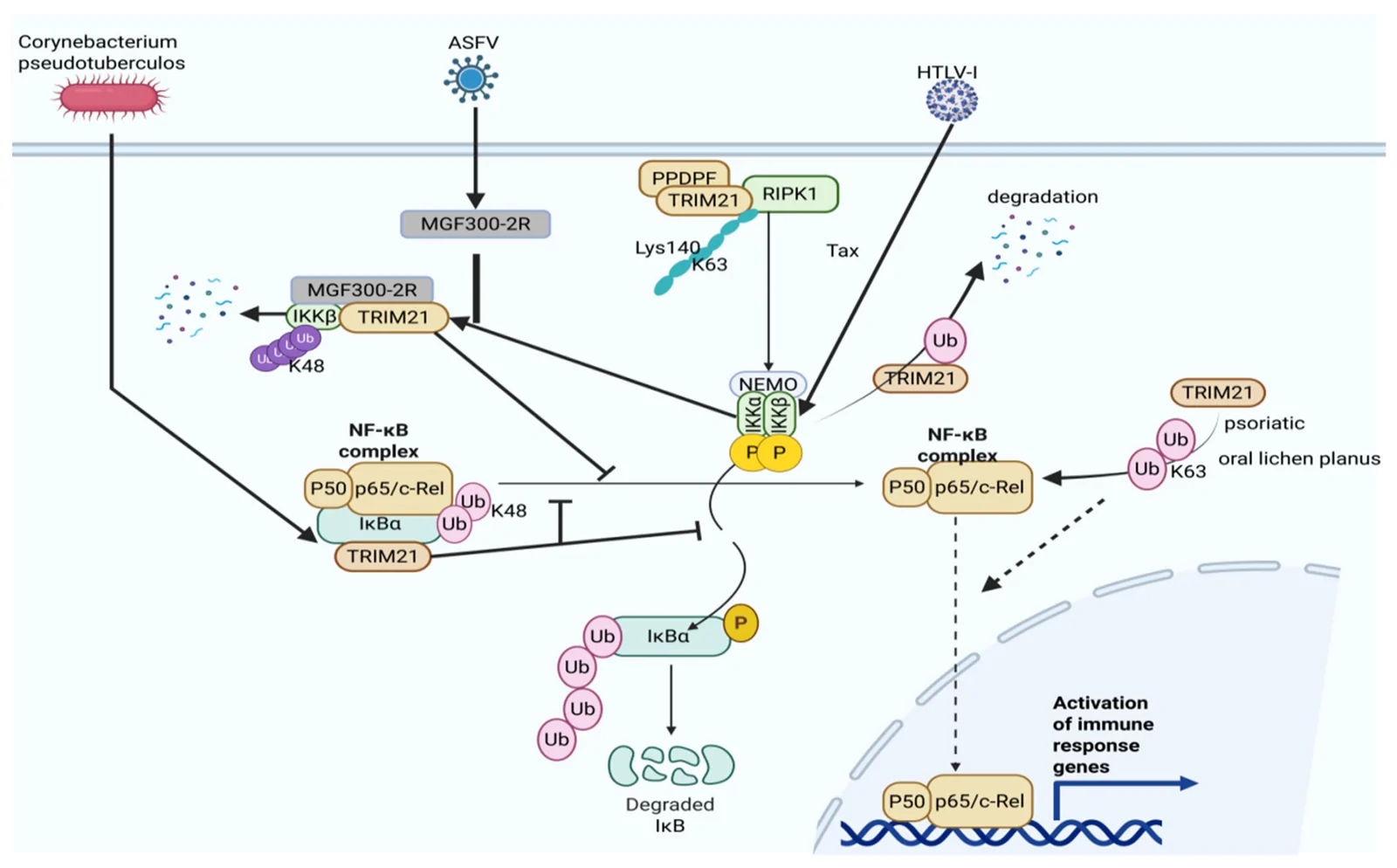
TRIM21 regulates NF-κB signaling during viral infection and inflammatory diseases. TRIM21 bidirectionally regulates NF-κB by ubiquitinating up-(RIPK1, IKKβ) and downstream (IκBα, p65) targets. ASFV MGF300-2R recruits TRIM21 to induce K48/K27-linked ubiquitination and autophagic degradation of IKKβ, suppressing pro-inflammatory cytokines. During HTLV-1 infection, TRIM21 mediates monoubiquitination and autophagic downregulation of active IKKβ, thereby suppressing Tax-induced NF-κB activation. Conversely, TRIM21-catalyzed K63-linked ubiquitination of p65 enhances its activation and nuclear translocation, thereby increasing inflammatory signaling. The figure was created using BioRender (BioRender.com).

The NF-κB complex is typically a homo- or heterodimer composed of Rel-family proteins, including p65 (RelA), RelB, c-Rel, p50 (NFκB1), and p52 (NFκB2). NF-κB complex is normally retained in the cytoplasm by IκB proteins, while viruses or pro-inflammatory cytokines activate the IKK complex (IKKα/IKKβ/NEMO), which phosphorylates IκBα, leading to its degradation and subsequent nuclear translocation of NF-κB, which then regulates the expression of genes involved in immune responses, inflammation, cell survival, and apoptosis [10]. TRIM21 has been confirmed to positively or negatively affect NF-κB activation by modulating the ubiquitination of key adaptor proteins, depending on cell type, interacting partner, viral infection, and disease state (Figure 2).

During vesicular stomatitis virus (VSV) infection, TRIM21 silencing significantly reduces p65 phosphorylation, indicating a positive regulatory role for TRIM21 in virus-induced NF-κB signaling [11]. In human T-cell leukemia virus type 1 (HTLV-1)-induced adult T-cell leukemia (ATL), persistent NF-κB activation driven by the viral oncogene Tax is considered a key pathogenic factor (Figure 2). Tax expression promotes IKKβ phosphorylation to chronically activate NF-κB signaling [12]. By contrast, TRIM21-mediated monoubiquitination and autophagic degradation of IKKβ significantly inhibit Tax-induced NF-κB activation [12]. African swine fever virus (ASFV)-encoded MGF300-2R protein recruits TRIM21 to catalyze K48- and K27-linked polyubiquitination of IKKβ, promoting its selective autophagic degradation (Figure 2). This process dampens p65-mediated production of pro-inflammatory cytokines (e.g., IL-1β, TNF-α), thereby enhancing viral immune evasion in macrophages [13]. These findings reveal the “MGF300-2R–TRIM21–IKKβ” axis as a novel mechanism by which ASFV suppresses host inflammatory responses.

Beyond its effects on upstream regulators, TRIM21 can directly induce K63-linked ubiquitination of specific NF-κB pathway components. The receptor-interacting serine/threonine-protein kinase 1 (RIPK1) acts as a scaffolding platform that undergoes K63-linked ubiquitination to recruit and activate the IKK complex, thereby promoting canonical NF-κB activation to control inflammatory signaling [14]. Recently, the pancreatic progenitor cell differentiation and proliferation factor (PPDPF) was shown to recruit TRIM21 to RIPK1, where TRIM21 catalyzes K63-linked ubiquitination at lysine 140 of RIPK1 (Figure 2). This modification mimics the RIPK1 K63-ubiquitinated state mediated by TRAF2/cIAP in TNF receptor signaling, stabilizing RIPK1 and recruiting the downstream NEMO/IKK complex, thereby enhancing NF-κB activation [15]. Notably, PPDPF overexpression specifically enhances TRIM21-mediated RIPK1 K63-ubiquitination to suppress excessive cell death via accelerated NF-κB activation, underscoring the crucial role of the PPDPF–TRIM21–RIPK1 complex in inflammatory responses and cell survival [15]. However, the involvement of this complex in viral infection remains unclear and warrants further investigation.

In addition to its effects on adaptor proteins and kinase complexes, TRIM21-mediated modification of core NF-κB components contributes to pathway regulation. In Corynebacterium pseudotuberculosis-infected macrophages, binding of TRIM21 to IκBα via its C-terminal PRY/SPRY domain is associated with IκBα stabilization and sustained suppression of NF-κB activation. In the absence of TRIM21, IκBα is more readily phosphorylated by IKKβ, leading to rapid degradation and lower IκBα levels, which results in an increase in the nuclear translocation of p65. Consequently, NF-κB-dependent inflammatory cytokines, including IL-1α, IL-1β, IL-6, and TNF-α, are significantly elevated in TRIM21-deficient macrophages and in mice compared with wild-type controls. Mechanistically, TRIM21 promotes K48-linked ubiquitination of IκBα, rather than K63-linked polyubiquitination [16]. TRIM21-mediated regulation reduces IκBα phosphorylation, thereby delaying its degradation and increasing its stability [16], but it is not uniformly inhibitory; in some disease contexts, it enhances NF-κB/p65 signaling in non-immune inflammatory cells (Figure 2). In psoriasis-like inflammation, patients exhibit significantly elevated TRIM21 levels in epidermal keratinocytes [17]. TRIM21 directly binds to the NF-κB p65 subunit in the cytoplasm, inducing its K63-linked polyubiquitination. Ubiquitinated p65 subsequently interacts with the IKK complex and undergoes phosphorylation-dependent activation, promoting p65 nuclear translocation and pro-inflammatory gene transcription [17]. Recently, similar mechanisms have been reported in infiltrating CD3+ T cells of oral lichen planus (OLP) [18]. However, it is not known whether these mechanisms exist in viral infections or other autoimmune diseases.

Collectively, TRIM21 exerts dual regulatory roles on NF-κB components. This dual regulatory strategy enables TRIM21 to play a balancing role across different pathological conditions [19]. Therefore, a deeper understanding of how TRIM21 specifically regulates the NF-κB pathway in different cells and environments is crucial for elucidating the pathogenesis of autoimmune diseases and chronic infections and may inform the development of precise intervention strategies targeting TRIM21.

## 3. Regulation of TRIM21 in PRR-Mediated Interferon Response Pathways

### 3.1. TRIM21 Regulates TLR-Mediated Innate Immune Signaling

TLRs recognize PAMPs on invading pathogens and activate the type I IFN and NF-κB signaling pathways to drive innate immune defense. In addition to the aforementioned regulation of the NF-κB pathway, previous studies have shown that TRIM21 mainly targets different IRF molecules to regulate the TLR-mediated IFN-I responses.

**Figure 3:**
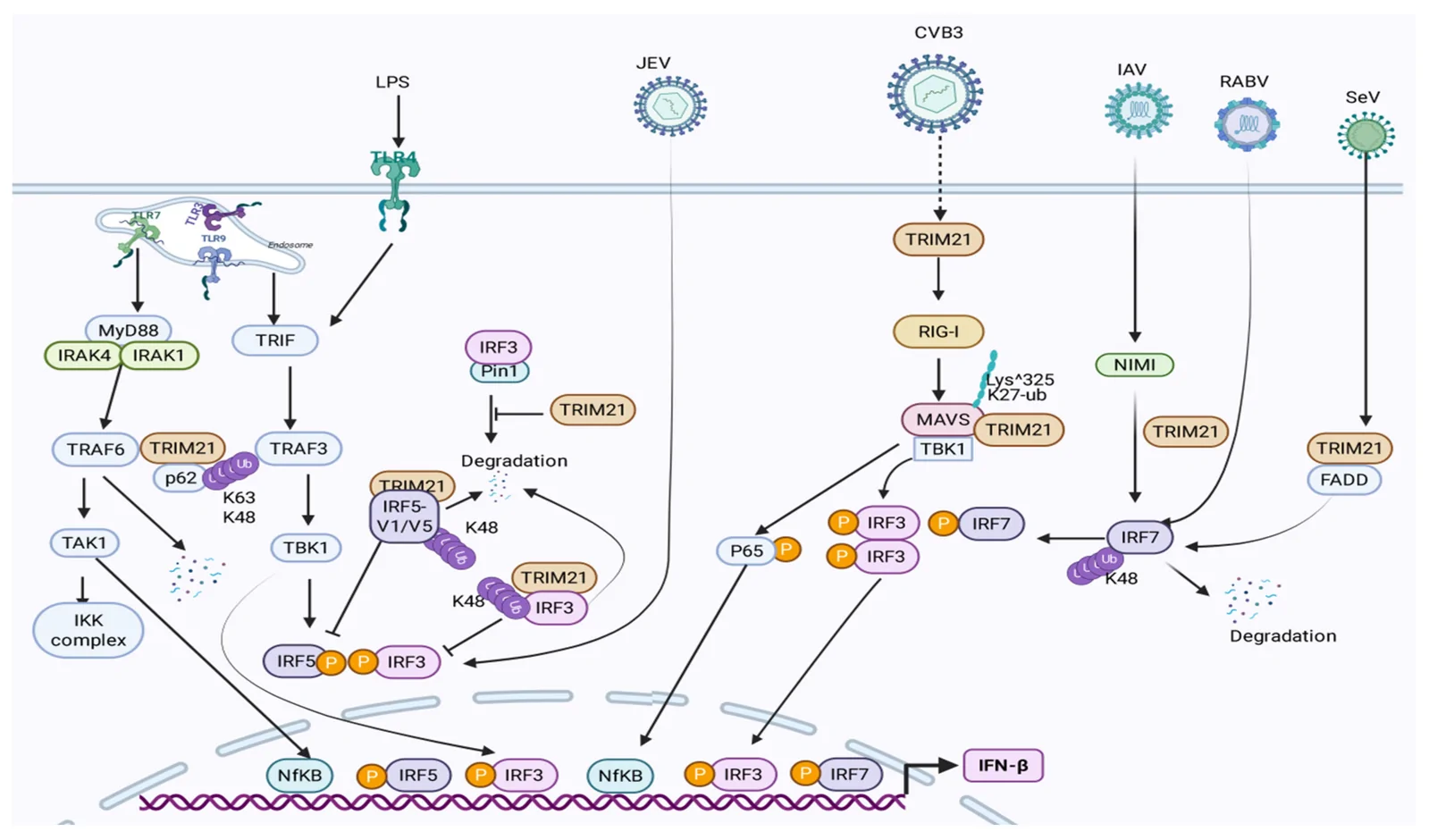
TRIM21’s dual regulatory role in the RNA virus-induced type I IFN pathway. Positive regulation: In cardiomyocytes infected with positive-strand RNA viruses (e.g., CVB3), TRIM21 binds MAVS via its PRY-SPRY domain and catalyzes K27-linked ubiquitination at MAVS Lys325, preventing MAVS degradation and recruiting TBK1 to activate IRF3, thereby enhancing IFN-β expression and limiting viral replication. Negative regulation: In host cells infected with negative-strand RNA viruses (e.g., IAV, RABV), TRIM21 is recruited via host factors (NMI/FADD) to IRF7 and triggers K48-linked polyubiquitination, leading to proteasomal degradation of IRF7, suppressing type I interferon production and promoting viral replication. The figure was created using BioRender (BioRender.com).

TRIM21 acts as a dual modulator of IRF3 function. Early studies found that TRIM21, depending on its C-terminal PRY/SPRY domain, directly binds to IRF3, thereby interfering with the interaction between IRF3 and Pin1 (peptidyl-prolyl cis/trans isomerase, NIMA-interacting 1) [20]. This disruption prevents premature ubiquitination and degradation of activated IRF3, which does not require the E3 ligase activity of TRIM21 (Figure 3). During Japanese encephalitis virus (JEV) infection of human microglial cells, TRIM21 expression is markedly increased and negatively regulates the type I interferon response by suppressing IRF3 phosphorylation and subsequent IFN-β production [21]. The E3 ubiquitin ligase activity of TRIM21 catalyzes K48-linked polyubiquitination of IRF3, resulting in its proteasomal degradation. This modification forms a negative-feedback mechanism that attenuates the IFN-I pathway [22]. These studies indicate that TRIM21-mediated regulation of IFN-I production through IRF3 may vary spatiotemporally across different viral infections.

TRIM21 also exerts a negative modulatory effect on IRF5. In TRIM21-deficient mice, abnormal accumulation of IRF5 is accompanied by increased IL-6 and autoantibody production and SLE-like autoimmune pathology. Following TLR7/9 activation, IRF5 drives the expression of pro-inflammatory cytokines, whereas TRIM21-mediated K48-linked polyubiquitination promotes its proteasomal degradation. This regulation is isoform-specific and depends on the presence of a PEST sequence, rendering IRF5 V1 and V5, but not V2 or V3, susceptible to TRIM21-mediated degradation. Thus, selective turnover of IRF5 isoforms provides a mechanism through which TRIM21 limits excessive TLR7/9-driven inflammation and contributes to immune homeostasis [23].

### 3.2. RIG-I-like Receptor Pathway: Function of TRIM21 in RNA Viral Infection

The RLR pathway is a critical mechanism by which cells recognize RNA viruses and induce IFN-I responses. Depending on the viral species and cell type, TRIM21 exerts dual regulatory effects on RLR-mediated antiviral IFN responses (Table 1) [24,25,26,27].

TRIM21 expression is upregulated during the early stage of CVB3 infection in mouse cardiomyocytes. TRIM21 overexpression significantly reduced viral titers and inflammatory damage in the myocardium and pancreas, whereas TRIM21 deficiency led to decreased IFN-β production, aggravated viral replication, and increased tissue damage [24]. The PRY-SPRY domain of TRIM21 directly interacts with MAVS and catalyzes its K27-linked polyubiquitination at lysine 325 (K325), preventing its degradation and enhancing TBK1 recruitment to the MAVS complex (Figure 3). Beyond CVB3, TRIM21 has also been identified as an IFN-responsive restriction factor against the flaviviruses ZIKV and LGTV, although the underlying molecular mechanism remains to be elucidated [28].

TRIM21 typically acts as a negative regulator of the IFN response in respiratory and neurotropic negative-strand RNA viral infections. Fas-associated death domain (FADD) protein was initially confirmed to interact with TRIM21, enhancing its E3 ligase activity. Formation of a TRIM21–FADD complex promotes K48-linked ubiquitination of IRF7, resulting in impaired IRF7 activation and phosphorylation and increased proteasomal degradation [25]. Recent evidence indicates that TRIM21 attenuates antiviral responses in human lung epithelial cells during IAV infection. IAV infection upregulates the N-myc and STAT interactor (NMI), which serves as a molecular bridge to recruit TRIM21 to IRF7 [26]. Experimental evidence confirms that TRIM21 knockout attenuates NMI overexpression-induced polyubiquitination of IRF7, prevents its degradation, and consequently enhances IAV-induced IFN signaling while reducing viral titers. Consistently, TRIM21 expression is also significantly elevated during rabies virus (RABV) infection, while TRIM21 knockout leads to increased IFN-I secretion and suppressed viral replication [27]. Mechanistically, TRIM21 catalyzes K48-linked polyubiquitination of IRF7, triggering its proteasomal degradation (Figure 3). Collectively, these findings indicate that TRIM21 negatively regulates antiviral immunity via the IRF7-IFN axis, paradoxically becoming a host factor exploited by viruses.

### 3.3. cGAS-STING Pathway: Function of TRIM21 in DNA Viral Infection

**Figure 4:**
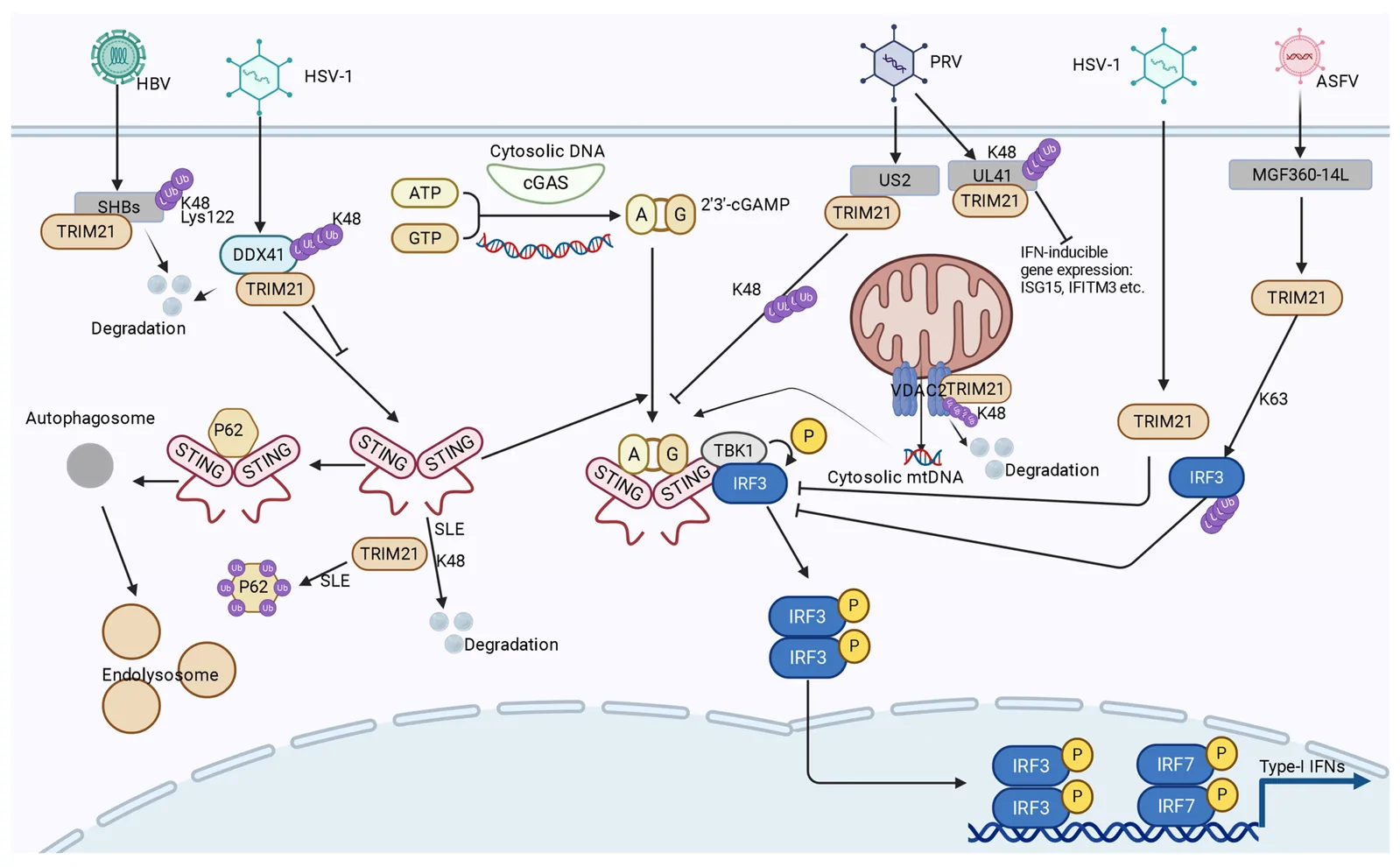
TRIM21 regulates the cGAS-STING pathway in DNA virus-infected cells. Positive regulation: In systemic lupus erythematosus (SLE), TRIM21 catalyzes K63-linked ubiquitination of p62/SQSTM1, blocking p62-mediated autophagic degradation and stabilizing STING to amplify IFN-β signaling. Negative regulation in viral infection and tumors: In DNA viral infection, TRIM21-mediated ubiquitination provides negative feedback: ASFV MGF360-14L recruits TRIM21 to induce K63-linked ubiquitination and proteasomal degradation of IRF3; HSV-1 upregulates TRIM21 to catalyze K48-linked ubiquitination of DDX41, degrading this DNA sensor; and PRV US2 recruits TRIM21 to conjugate K48-linked chains on STING, causing its proteasomal degradation and suppressing IFN production. In nasopharyngeal carcinoma, TRIM21-mediated K48 ubiquitination promotes VDAC2 degradation, reducing mitochondrial DNA release and cGAS-STING activation. Additional DNA-virus-related effects include TRIM21-mediated K48-linked proteasomal degradation of PRV UL41 and HBV SHBs at Lys122. In SLE, TRIM21 can also directly promote ubiquitin–proteasome-dependent STING degradation, opposing its indirect p62/SQSTM1-mediated STING-stabilizing effect. The figure was created using BioRender (BioRender.com).

Growing evidence suggests that STING plays a central role in the immune response to viral infection and tumorigenesis as well as in autoimmune diseases. Although cGAS is the major DNA sensor that activates the STING-TBK1-IRF3 axis, there is no direct evidence indicating that TRIM21 modifies cGAS. Beyond the canonical cGAS-STING axis, STING exhibits alternative activation mechanisms via cytosolic DNA sensors, including the DNA-dependent activator of interferon-regulatory factors (DAI), IFNγ-inducible protein 16 (IFI16), and DEAD-Box Helicase 41 (DDX41) [49,50,51]. In a corneal epithelial infection model with HSV-1, DDX41 binds to the HSV-1 genome and recruits TBK1 via STING to activate IRF3 [31]. TRIM21 was also significantly upregulated and inhibited IFN-I production in HSV-1-infected cells [32]. Mechanistically, DDX41 is a direct substrate of TRIM21, whereas IFI16 and other DNA sensors show negligible effects [31]. TRIM21 engages DDX41 via its PRY/SPRY domain and catalyzes the K48-linked polyubiquitination and proteasomal degradation of DDX41, thereby abrogating the transmission of upstream activation signals to STING (Figure 4).

TRIM21 is an important E3 ligase involved in suppressing innate immune signaling by some DNA viruses (Table 1). TRIM21 ablation significantly enhanced the antiviral activity of the STING–IRF3 axis, while viral titers were decreased in porcine pseudorabies virus (PRV)-infected mice or HSV-1-infected cells [32,33]. The envelope protein US2 of PRV can directly interact with the ligand-binding domain (LBD) of STING and recruit TRIM21 to catalyze its K48-linked polyubiquitination and degradation [33]. Interestingly, an opposite outcome of TRIM21-mediated activity is observed for another PRV protein. A recent study showed that the PRV virion host shutoff protein UL41 suppresses IFN signaling through the JAK/STAT pathway, whereas TRIM21 binds UL41 through its PRY/SPRY domain and promotes its K48-linked ubiquitination and proteasomal degradation. Thus, PRV provides a notable example in which TRIM21-mediated ubiquitination can either contribute to suppression of innate sensing or promote degradation of a viral immune antagonist, depending on the viral component involved [34]. The ASFV-encoded MGF360-14L protein, an immunosuppressive factor, was identified as recruiting TRIM21 to inhibit type I IFN production [35]. Specifically, MGF360-14L enhances TRIM21-mediated K63-linked polyubiquitination of IRF3, promoting its proteasomal degradation [35]. Moreover, the ASFV MGF300-2R protein recruits TRIM21, resulting in K48- and K27-linked polyubiquitination of IKKβ and TOLLIP-mediated selective autophagic degradation, thereby attenuating NF-κB-driven inflammatory signaling [13]. In addition to its regulation of host innate immune components, TRIM21 can directly restrict a DNA virus by targeting a viral protein. TRIM21 restricts HBV by interacting with SHBs and promoting its K48-linked polyubiquitination at Lys122 and subsequent proteasomal degradation, thereby reducing SHB abundance and viral particle production; this process is counteracted by OTUD4 [36].

TRIM21-mediated regulation also affects mitochondrial DNA (mtDNA)-induced innate immunity (Figure 4). The voltage-dependent anion channel 2 (VDAC2) is an established regulator of mitochondrial permeability, metabolite exchange, and mtDNA release. The NS4B protein of dengue virus (DENV) binds to VDAC2, alters mitochondrial morphology, and weakens innate immune responses; downregulating VDAC2 significantly reduces DENV replication [52]. The VP5 protein of infectious bursal disease virus (IBDV) induces apoptosis by binding to VDAC2, thereby affecting viral replication [53]. Recently, TRIM21-mediated K48-linked ubiquitination was shown to promote VDAC2 degradation in nasopharyngeal carcinoma [41]. This degradation reduces mtDNA release and suppresses the cGAS-STING signaling pathway, leading to decreased IFN expression [41]. Therefore, it is worth considering whether the TRIM21-mediated VDAC2 degradation mechanism might suppress the virus-induced mtDNA release and activation of the cGAS-STING-IFN-I axis, thereby achieving immune evasion [54].

Recently, TRIM21 has been shown to regulate STING stability through apparently distinct mechanisms in SLE. One study demonstrated that TRIM21 catalyzes K63-linked ubiquitination of p62/SQSTM1, thereby disrupting the p62–STING interaction, limiting autophagic degradation of STING, and consequently enhancing STING stability and downstream type I IFN signaling [39]. By contrast, another recent study reported that TRIM21 can directly interact with STING and facilitate its ubiquitin–proteasome-dependent degradation, thereby restraining STING activation and type I IFN production. TRIM21 therefore exerts differential regulation of STING, and whether this plasticity is exploited by viruses remains an important question for future investigation [40].

## 4. TRIM21-Mediated Selective Autophagy in Viral Manipulation of Host Protein Responses

**Figure 5:**
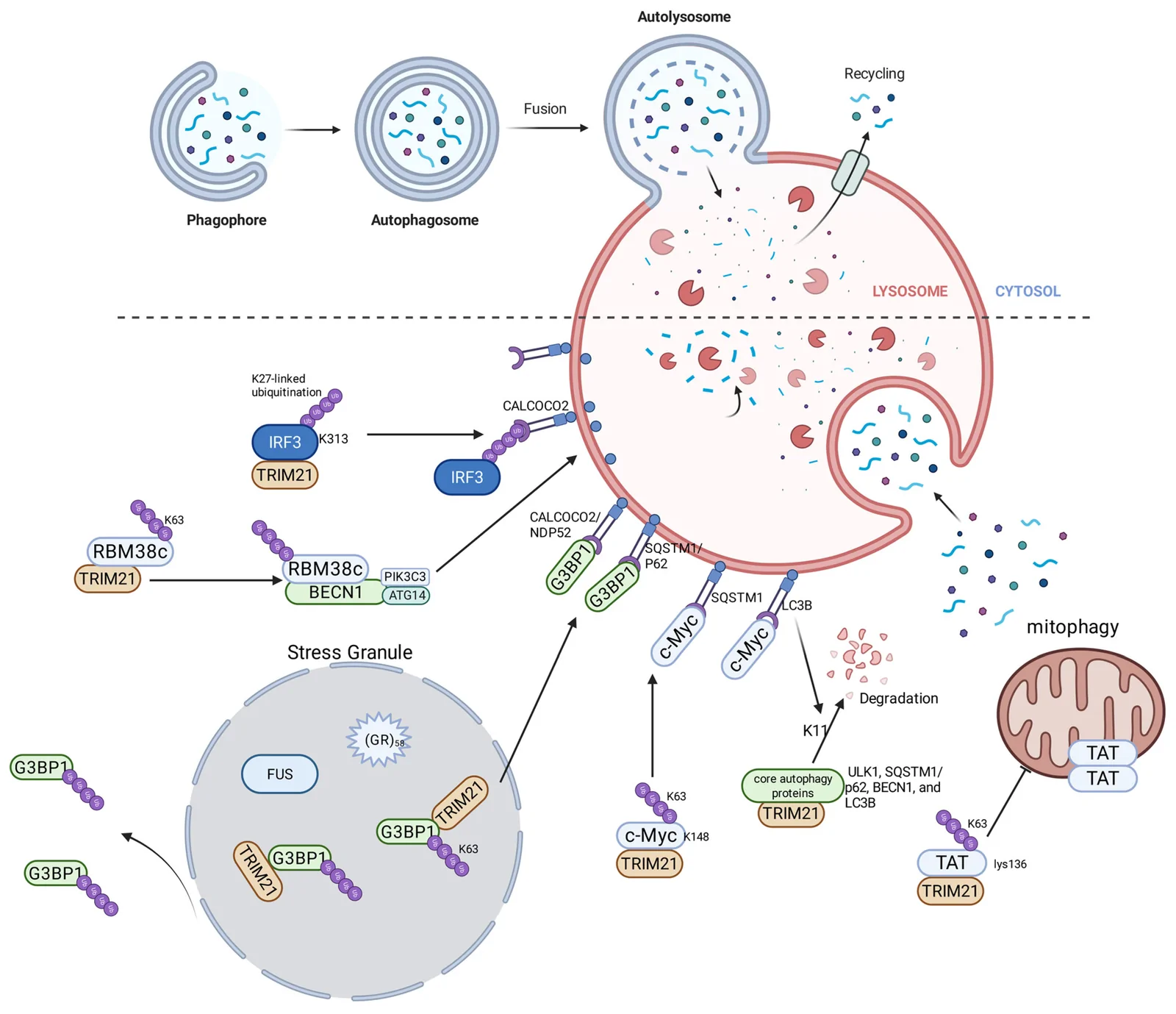
TRIM21-mediated selective autophagic pathway in antiviral defense or diseases. TRIM21-mediated ubiquitination and adaptor activity contribute to selective autophagic elimination of virus-associated or aggregated host factors. K27-linked ubiquitination of IRF3 by TRIM21 enables NDP52-mediated autophagic degradation, thereby limiting excessive type I interferon signaling. TRIM21-mediated K63-linked ubiquitination of G3BP1 within virus-induced stress granules promotes p62/NDP52-dependent autophagic clearance of the granule complex. In cancer models, TRIM21 promotes autolysosomal degradation of the host proto-oncoprotein c-Myc. In septic acute lung injury, TRIM21 promotes K11-linked degradation of ULK1, p62, BECN1, and LC3B, suppressing autophagy and promoting M1 polarization. Conversely, K63-linked ubiquitination of RBM38c enhances BECN1 binding and autophagy initiation. The figure was created using BioRender (BioRender.com).

Selective autophagy relies on defined cargo-recognition mechanisms that couple ubiquitin tagging to receptor-mediated delivery of substrates into the autophagosome-lysosome pathway [55]. TRIM21-mediated ubiquitination has recently been shown to mark specific substrates for autophagic degradation. In concert with E2 enzymes, TRIM21 catalyzes assembly of K63- or K27-linked ubiquitin chains at specific substrate sites, which serve as selective autophagic signals [56]. Upon SeV infection, TRIM21 binds to dimerized IRF3, particularly catalyzing the K27-linked ubiquitination of IRF3 at K313 (Figure 5). Silencing TRIM21 reduces K27 ubiquitination and stabilizes IRF3. Mechanistically, the autophagy receptor NDP52 specifically recognizes the K27-linked ubiquitination of IRF3 for autophagic degradation, whereas SQSTM1/p62 does not [37]. Hence, the TRIM21–IRF3–NDP52 pathway may limit excessive IFN-I signaling in virus-infected cells.

G3BP1, a core stress granule (SG) protein, mediates host innate immunity and viral proliferation. As an antiviral scaffold, G3BP1 recruits viral RNA, RIG-I, and PKR into antiviral SGs, thereby facilitating non-self RNA recognition and downstream IFN-β production [57,58]. Moreover, G3BP1 can stabilize RIG-I by inhibiting RNF125-mediated K48-linked ubiquitination and proteasomal degradation [59]. However, infectious bursal disease virus (IBDV) infection upregulates G3BP1 expression and triggers characteristic SG formation, thereby facilitating viral replication [60]. Conversely, some viruses have recently been shown to employ strategies to suppress SG formation. SARS-CoV-2 nucleocapsid protein recruits G3BP1 to viral replication membrane vesicles, blocking cytoplasmic SG assembly [61]. TRIM21 has been identified as a direct interactor of aggregated G3BP1, inducing its K63-linked ubiquitination under oxidative stress or viral infection (Figure 5). Ubiquitinated G3BP1 is recognized by the autophagic receptors NDP52 and p62, bridging G3BP1-containing SG aggregates into LC3-positive autophagosomes [38].

c-Myc, an important cancer-related protein, substantially increases the risk of cellular carcinogenesis and sustained viral replication (Figure 5). EBV-induced c-Myc overexpression can suppress the tumor suppressor p53, thereby promoting cell proliferation in EBV-associated tumors [62]. The oncoproteins E6/E7 of high-risk human papillomavirus (HPV) also abnormally enhance c-Myc transcriptional activity and stability, leading to cell cycle disorders and malignant proliferation tendencies in virus-infected cells [63]. TRIM21 binding to c-Myc was recently shown to promote K63-linked ubiquitination at K148 and subsequent autolysosomal degradation [42]. Similarly, TRIM21-catalyzed K63-linked ubiquitination of the pro-metastatic enzyme tyrosine aminotransferase (TAT) disrupts its dimerization and mitochondrial localization, ultimately reducing tumor cell invasiveness and hepatic metastasis [43]. Given that EBV and HPV infections are known to induce abnormal c-Myc activation, it is plausible that TRIM21-mediated selective autophagic degradation of c-Myc serves as a regulatory mechanism to counteract viral subversion of host proliferative signaling. Hence, it is worth exploring whether TRIM21 could effectively prevent EBV and HPV replication and virus-induced aberrant proliferation signaling by reducing intracellular c-Myc.

Beyond selective ubiquitination of individual substrates, TRIM21-mediated ubiquitination also directly modifies components of the autophagy machinery. In septic acute lung injury, TRIM21 in macrophages promotes K11-linked ubiquitination and proteasomal degradation of ULK1, SQSTM1/p62, BECN1, and LC3B, thereby suppressing autophagy and enhancing pro-inflammatory M1 polarization [44]. Conversely, following DNA damage, non-degradative TRIM21-mediated K63-linked ubiquitination of RBM38c at Lys35 enhances its interaction with BECN1 and facilitates autophagy initiation [45].

## 5. Future Perspectives

Ubiquitination, as a dynamic post-translational modification, plays a significant role in modulating the function of substrate proteins and is deeply involved in precisely regulating the transmission and intensity of innate immune signals. This process not only involves classical protein degradation but also exerts a core function at multiple levels of the immune response by altering the stability, activity, localization, and interactions of proteins. TRIM21, as an E3 ligase targeting various substrate proteins, serves as a pivotal and multifaceted regulator of the innate immune response in viral infections and autoimmune diseases. Many viruses, such as ASFV, HSV-1, and PRV, have evolved to recruit or upregulate TRIM21 to degrade crucial immune signaling proteins, thereby dampening host antiviral responses [32,33,35]. Hence, TRIM21 presents significant “double-edged sword” characteristics under different pathological conditions in regulating IFN and NF-κB signaling. Notably, such bidirectional regulation is shared by other TRIM-family members identified through overexpression or candidate-driven screens, but these approaches have inherent limitations. In the future, high-throughput perturbation strategies such as Perturb-seq, combined with primary and immortalized cells, viral infection models, and single-cell analyses, may help identify new TRIM21 substrates and partners while distinguishing conserved functions from cell- or context-specific effects. Such approaches will provide a stronger framework for understanding bidirectional NF-κB regulation and evaluating TRIM21-dependent ubiquitination networks as therapeutic targets.

The biochemical properties of TRIM21 offer substantial potential for therapeutic degradation of intracellular pathogen antigens and specific host proteins. As an intracellular Fc receptor, TRIM21 links innate and adaptive immunity through Fc binding [29,30]. Binding of antibody-coated virions to TRIM21 results in intracellular neutralization, providing an additional layer of defense against invading pathogens. TRIM21-mediated selective autophagy also contributes to cellular homeostasis through lysosomal degradation of virus-associated host factors and selected proteins such as G3BP1 and c-Myc. Moreover, PRLX-93936 and BMS-214662 were recently confirmed as molecular glues that directly target TRIM21 to induce degradation of nucleoporin proteins and inhibit nuclear trafficking [48]. Clinically, the “Trim-Away” technology relies on endogenous TRIM21-mediated antibody-dependent degradation of intracellular proteins [46]. Recent proof-of-concept studies demonstrated its efficacy against ASFV: nanobodies targeting viral capsid proteins p30, p54, and p72, when delivered into infected cells, recruited TRIM21 to induce proteasomal and lysosomal degradation of viral proteins, significantly reducing viral yield [47].

## 6. Conclusions

In summary, elucidating the dynamic regulatory mechanisms of TRIM21 not only reconciles its seemingly paradoxical functions but also opens new avenues for precise therapeutic modulation of antiviral and immune-mediated diseases. TRIM21 may serve as a model for how immune balance can be finely tuned to favor host defense and homeostasis in the future.

## Figures and Tables

**Table 1:** Reference-supported mechanisms of TRIM21 in viral infection, innate immunity, selective autophagy, and disease contexts.

Functional Category	Virus/Disease Model	Cell Type/System	TRIM21 Substrate/Partner	Ub LINKAGE/Modification	Degradation/Regulatory Route	Biological Outcome	Affected Pathway	Ref.
NF	HTLV-1/Tax	T cells	IKKβ	Ub	Autophagic downregulation of active IKKβ	Suppresses Tax-induced NF-κB activation	NF-κB	[12]
NF	ASFV MGF300-2R	Macrophages	IKKβ	K48; K27	TOLLIP-mediated selective autophagy	Reduces NF-κB-driven inflammatory cytokines	NF-κB	[13]
NF	HCC/inflammatory signaling	Hepatoma cells	RIPK1 (K140)	K63	Non-degradative signaling activation	Promotes NEMO/IKK recruitment and NF-κB activation	NF-κB	[15]
NF	C. pseudotuberculosis infection	Macrophages	IκBα	K48	Maintains IκBα stability; restrains degradation	Limits p65 nuclear translocation and cytokines	NF-κB	[16]
NF	Psoriasis model	Keratinocytes	p65/RelA	K63	Non-degradative activation	Promotes p65 phosphorylation and nuclear translocation	NF-κB	[17]
NF	Oral lichen planus	T cells	p65/RelA	K63	Non-degradative activation	Enhances inflammatory cytokine production	NF-κB	[18]
+	VSV/RNA virus-triggered response	Cells	MAVS	K27	Signaling enhancement; no degradation	Promotes antiviral innate immune response	RLR–MAVS–TBK1–IRF3	[11]
+	CVB3 infection	Cardiomyocytes	MAVS (K325)	K27	Stabilization/signal enhancement	Increases TBK1 recruitment and IFN-β; restricts virus	RLR–MAVS–TBK1–IRF3	[24]
+	ZIKV/LGTV infection	Cells	Not defined	Not defined	IFN-responsive restriction; mechanism unresolved	Restricts flavivirus infection	Antiviral IFN response	[28]
+	Antiviral response	Cells	IRF3	No Ub	Disrupts IRF3–Pin1 interaction; stabilizes IRF3	Sustains IRF3 activation and IFN response	TLR/RLR–IRF3	[20]
+	Antibody-bound virions/pathogens	Cells	Fc-bound cargo/TRIM21	Ub; ubiquitin-dependent regulation	Proteasomal degradation and intracellular neutralization	Links humoral immunity with intracellular defense	Cytosolic Fc receptor immunity	[29,30]
−	Virus-induced IFN response	Cells	FADD–TRIM21–IRF7	K48	Proteasomal degradation	Suppresses virus-induced IFN-I production	RLR–IRF7	[25]
−	IAV infection	Lung epithelial cells	NMI–TRIM21–IRF7	K48	Proteasomal degradation	Inhibits IFN signaling and facilitates IAV replication	RLR–IRF7	[26]
−	RABV infection	Cells	IRF7	K48	Proteasomal degradation	Promotes RABV replication via IFN suppression	RLR–IRF7	[27]
−	HSV-1 epithelial keratitis	Epithelial cells	DDX41	K48	Proteasomal degradation	Blocks DDX41–STING signaling and aggravates disease	DDX41–STING–IRF3	[31,32]
−	PRV infection/US2	Cells	STING	K48	Proteasomal degradation	Antagonizes cGAS–STING antiviral signaling	cGAS–STING	[33]
+	PRV infection/UL41	Cells	UL41	K48	Proteasomal degradation	Removes viral IFN antagonist; restores antiviral signaling	JAK/STAT–IFN	[34]
−	ASFV MGF360-14L	Cells	IRF3	K63	Proteasomal degradation in reported context	Reduces type I IFN production	STING/TBK1–IRF3	[35]
+	HBV infection	Cells	SHBs (K122)	K48	Proteasomal degradation	Reduces SHB abundance and viral particle production	HBV restriction	[36]
−	TLR7/autoimmunity	Immune cells	IRF5 isoforms	K48	Proteasomal degradation	Limits IRF5 accumulation and autoimmunity	TLR7/9–IRF5	[23]
−	SeV-triggered signaling	Cells	IRF3 (K313)	K27	NDP52-mediated selective autophagy	Terminates excessive IFN-I signaling	RLR–IRF3	[37]
−	Stress/viral SG context	Cells	G3BP1	K63	p62/NDP52-mediated selective autophagy	Clears stress granules; regulates innate responses	Stress granule homeostasis	[38]
+	SLE	Immune cells	p62/SQSTM1 (indirectly STING)	K63	Blocks p62-mediated autophagic degradation of STING	Stabilizes STING and enhances IFN-I signaling	STING–IFN-I	[39]
−	SLE	Immune cells	STING	Ub; linkage not specified	Ubiquitin–proteasome-dependent degradation	Restrains STING activation and type I IFN production	STING–IFN-I	[40]
−	Nasopharyngeal carcinoma	Tumor cells	VDAC2	K48	Proteasomal degradation	Reduces mtDNA release and dampens cGAS–STING activation	mtDNA–cGAS–STING	[41]
H	Colorectal cancer	CRC cells	c-Myc	K63	Autolysosomal degradation	Suppresses oncogenic signaling; increases drug sensitivity	Oncogenic signaling	[42]
H	Gallbladder cancer	Cancer cells	TAT	K63	Autolysosomal degradation	Suppresses liver metastasis	Metastasis/metabolism	[43]
−	Septic acute lung injury	Macrophages	ULK1; SQSTM1/p62; BECN1; LC3B	K11	Proteasomal degradation	Suppresses protective autophagy; promotes pro-inflammatory M1 polarization	Autophagy/inflammation	[44]
+	DNA damage	Cells	RBM38c (K35)	K63	Non-degradative; enhances RBM38c–BECN1 interaction	Promotes ATG14–PtdIns3K-C1 assembly and autophagy initiation	Autophagy initiation	[45]
H	ASFV nanobody TRIM-Away	Infected cells	ASFV p30, p54, p72	Ub; nanobody/antibody-directed TRIM21 recruitment	Proteasomal and lysosomal degradation	Reduces ASFV proteins and viral yield	Targeted antiviral degradation	[46,47]
H	TRIM21-targeting molecular glues	Cells	TRIM21-recruited nucleoporin targets	TRIM21-dependent ubiquitination; linkage not specified	Targeted protein degradation	Inhibits nuclear trafficking; supports TRIM21-based targeted degradation	Targeted protein degradation	[48]

Abbreviations: ASFV, African swine fever virus; CVB3, coxsackievirus B3; HCC, hepatocellular carcinoma; HSV-1, herpes simplex virus type 1; IAV, influenza A virus; PRV, pseudorabies virus; RABV, rabies virus; SeV, Sendai virus; SG, stress granule; SLE, systemic lupus erythematosus. Category: NF, regulator of NF-κB signaling; +, positive regulator; −, negative regulator; H, other immune homeostasis and disease extensions.

## Data Availability

No new data were created or analyzed in this study.
